# Patient-reported voice and swallowing outcomes and their impact on quality of life in the late postoperative period following total thyroidectomy

**DOI:** 10.1590/2317-1782/e20250387en

**Published:** 2026-07-31

**Authors:** Lívia Lima do Nascimento Silva, Vivian Lisboa de Lucena, Ana Flávia de Sales Cândido, Raphaela de Lima Cruz, Anna Alice Almeida, Leandro Pernambuco

**Affiliations:** 1 Curso de Graduação em Fonoaudiologia, Universidade Federal da Paraíba – UFPB - João Pessoa (PB), Brasil.; 2 Departamento de Fonoaudiologia, Hospital Napoleão Laureano – HNL - João Pessoa (PB), Brasil.; 3 Programa de Pós-graduação em Modelos de Decisão e Saúde – PPGMDS, Universidade Federal da Paraíba – UFPB - João Pessoa (PB), Brasil.; 4 Curso de Graduação em Fonoaudiologia, Universidade Federal de Pernambuco – UFPE - Recife (PE), Brasil.

**Keywords:** Thyroidectomy, Swallowing Disorders, Dysphonia, Postoperative, Quality of Life

## Abstract

**Purpose:**

To analyze self-assessment of voice and swallowing in the late postoperative period of total thyroidectomy and the impact of surgery on quality of life.

**Methods:**

This is a descriptive, observational, and cross-sectional study, approved by the Research Ethics Committee of the institution. Seventeen women aged between 32 and 58 years participated in the study, mostly married, with completed higher education, residing outside the metropolitan region, who underwent total thyroidectomy without cervical lymph node dissection, without adjuvant radiotherapy, and without postoperative speech therapy follow-up for at least 6 months. Demographic and clinical data were collected, as well as the results of the Thyroidectomy-Related Voice and Symptom Questionnaire (TVSQ) and the Thyroid-Related Patient-Reported Outcome - 39 (ThyPro-39) questionnaires. Data analysis was performed descriptively.

**Results:**

The median total TVSQ score was 26, with the highest scores found in the two cases that underwent cervical lymph node dissection. Domain 1 (vocal symptoms) of the TVSQ contributed most to the total score (48.52%). In domain 2 (oropharyngolaryngeal symptoms) of the TVSQ, scores were closer to the upper limit, representing a higher frequency of symptoms. The ThyPro-39 results revealed a greater impact of thyroidectomy on fatigue, anxiety, cognitive problems, emotion, and depression.

**Conclusion:**

Women undergoing total thyroidectomy present vocal and swallowing symptoms, with a predominance of vocal symptoms in the long term, associated with the negative impact of surgery on physical, emotional, and mental well-being. Furthermore, there is a compromise in quality of life regarding fatigue, emotional aspects, anxiety, cognitive problems, and depression.

## INTRODUCTION

Thyroidectomy is the most used surgical technique for conditions affecting the thyroid gland, totally or partially resecting it depending on the extent of the disease^(1)^. In many cases, these patients exhibit a range of symptoms affecting voice and swallowing^(2)^.

Changes in voice and swallowing may result from damage to the laryngeal nerves, particularly the recurrent laryngeal nerve (RLN) or the external branch of the superior laryngeal nerve (SLN)^(3)^. However, other possible etiologies are also observed for the condition known as post-thyroidectomy syndrome, such as trauma to the arytenoid cartilage; laryngotracheal fixation of the muscles involved due to postoperative adhesions; reduced laryngeal elevation; lesions of the extrinsic perithyroid nerve plexus; and edema and trauma to the cricothyroid muscle^(2,4)^.

Thus, studies demonstrate that thyroidectomy can impair the voice and compromise the functioning of the oropharyngeal-laryngeal system^(5,6)^. The most reported symptoms among the main ones are hoarseness, vocal fatigue, a deep voice, difficulty speaking loudly, a foreign body sensation in the pharynx, choking, throat clearing during swallowing, and a dry throat^(6)^.

Some studies report that vocal and swallowing changes may persist even long after total thyroidectomy^(3)^. A study aimed to develop validity evidence based on the internal structure of the Brazilian Portuguese version of a tool for early identification and monitoring of these symptoms; it found that most respondents were in the late postoperative period and still had significant complaints^(7)^.

In addition to voice and swallowing changes, problems affecting patients’ quality of life have been frequently reported^(8,9)^, such as fear of disease recurrence, poor sleep quality, job loss, sudden bouts of fatigue, physical exhaustion, fear of a second surgery, a globus sensation (lump in the throat), swallowing difficulties, and mental exhaustion^(8)^.

Thus, these complaints must be monitored and documented to track which signs and symptoms persist in the late postoperative period, which begins 6 months after the surgical procedure^(10)^.

Hence, this study aimed to analyze self-assessment of voice and swallowing in the late postoperative period following total thyroidectomy and their impact on quality of life in the same period.

## METHOD

This is a descriptive, observational, cross-sectional study approved by the Human Research Ethics Committee of the Health Sciences Center at the Federal University of Paraíba, Brazil, under approval number 6.018.537/2023. All participants were informed about the objectives, risks, and benefits of the study and signed an informed consent form. The study was conducted at the Napoleão Laureano Hospital in João Pessoa, Paraíba, Brazil, a nonprofit hospital maintained by a foundation, classified as a High-Complexity Oncology Care Center (CACON).

The inclusion criteria were individuals of both sexes, aged 18 to 59 years, who had undergone total thyroidectomy with or without neck dissection at least 6 months prior. The study excluded individuals who had previously undergone any other type of head and neck surgery, patients with tumor recurrence and/or distant metastases, individuals undergoing adjuvant therapy such as chemotherapy, radiotherapy, and iodine therapy at the time of data collection, patients with difficulty responding to simple commands or with cognitive or neurological impairments documented in medical records, observed by the researchers, or reported by companions.

An active search was initially conducted in the medical records of patients treated in the hospital’s Speech-Language Pathology department, and those meeting the inclusion criteria were selected to be invited via telephone contact. Additionally, patients present at the hospital for medical and/or speech-language appointments had their medical records reviewed and were invited to participate in the study if eligible.

The eligibility criteria identified 32 eligible individuals through medical record analysis, of whom 15 were excluded for not responding to contact or refusing to participate. Thus, the sample consisted of 17 individuals, all biological females, aged 32 to 58 years (mean: 46.64; SD: 8.65), who underwent total thyroidectomy between 2004 and 2022. Most participants were married (n = 12; 70.5%), had completed higher education (n = 7; 41.1%), and resided in cities outside the metropolitan area (n = 9; 52.9%), submitted to total thyroidectomy without neck dissection (n = 14; 82.3%), without adjuvant radioiodine therapy or radiation therapy (n = 11; 64.7%), and without prior speech and/or swallowing therapy (n = 14; 82.3%).

The data collection instruments were administered in two formats (in person or online, via Google Forms), including an eligibility checklist and a form for collecting demographic and clinical data, which supplemented the information previously gathered from medical records. Next, the Brazilian Portuguese versions of the Thyroidectomy-Related Voice and Symptom Questionnaire (TVSQ)^(11)^ and the Thyroid-Related Patient-Reported Outcome-39 (ThyPro-39br)^(12)^ were administered.

TVSQ has 20 questions on voice symptoms, swallowing, and cervicothoracic discomfort related to thyroidectomy. There are five response options for each item on a Likert-type scale, with values ranging from 0 to 4 (never, almost never, sometimes, almost always, and always), corresponding to the perceived frequency of the sign or symptom over the previous 30 days. This study analyzed TVSQ descriptively, considering the three domains identified by Cândido (2021)^(7)^ when analyzing the instrument’s structural validity: 1) vocal symptoms; 2) oropharyngeal-laryngeal symptoms; and 3) cervical and thoracic discomfort symptoms. The second TVSQ domain, called “oropharyngeal-laryngeal symptoms,” corresponds to what this study refers to as “swallowing symptoms”. The final TVSQ score is obtained by simply summing all items, ranging from 0 to 80; higher scores indicate a worse condition. The Brazilian Portuguese TVSQ version does not yet have defined cutoffs; therefore, the authors used two other indicators for interindividual comparisons in this descriptive study: 1) the domain’s relative score ([individual’s score in the domain / maximum score for the domain] × 100); and 2) the domain’s contribution ratio to the total score ([individual’s score in the domain / individual’s total score] × 100).

ThyPro-39 assesses and identifies self-perceived effects of thyroid diseases on quality of life. Its 39 questions are distributed across 13 categories, with responses rated on a 5-point Likert scale based on the patient’s perception over the previous 4 weeks (where 0 equals “not at all”, and 4 equals “very much”). The categories include four scales of physical symptoms; seven scales on well-being and physical, mental, and social functioning; one scale regarding appearance; and one item on the impact on overall quality of life. The results follow a specific transformation pattern that generates a score ranging from 0 to 100, distributed across each of the 13 scales of the instrument. Thus, the higher the score, the greater the impact on quality of life.

Data analysis was performed descriptively, using absolute and relative frequencies, and considering the mean, median, standard deviation, and interquartile range.

## RESULTS

The TVSQ results are shown in Table 1.

**Table 1:** Results of the Thyroidectomy-Related Voice and Symptom Questionnaire (TVSQ), adapted into Brazilian Portuguese, in individuals who underwent total thyroidectomy 6 or more months before (n = 17)

	**Mean**	**Median**	**Standard deviation**	**Minimum- maximum**	**Interquartile range(Q_1_-Q_3_)**
**Total score**	27.00	26.00	15.062	3-49	14.00-40.50
**Domain 1 (D1)** *Vocal symptoms (10 items)*	13.88	16.00	9.347	0-28	4.00-2.10
**Domain 2 (D2)** *Oropharyngeal-laryngeal symptoms (5 items)*	8.82	8.00	5.35	0-18	5.00-13.00
**Domain 3 (D3)** *Cervical and thoracic discomfort (5 items)*	4.29	3.00	3.73	0-12	1.50-7.50
**Relative D1 score** *(sum of D1/40 items)*	34.67	40.00	23.35	0-70	10.00-52.5
**Relative D2 score** *(sum of D2 items/20)*	44.11	40.00	26.76	0-90	25.00-65.00
**Relative D3 score** *(sum of D3/20 items)*	21.47	15.00	18.68	0-60	7.50-37.50
**Proportion of D1 in the total score** (sum of D1 items/total score)*100	49.05	48.98	21.87	0-85.71	36.66-66.67
**Proportion of D2 in the total score** (sum of D2 items/total score)*100	35.53	30.95	22.30	0-100	24.04-45.28
**Proportion of D3 in the total score (** sum of D3 items/total score)*100	15.41	15.38	10.78	0-33.33	6.60-24.95

The median total score was 26, with the two highest scores (49 and 48) belonging to two participants who underwent total thyroidectomy with neck dissection. The median normalized score reached 40% of the maximum possible score in “vocal symptoms” and “oropharyngeal-laryngeal symptoms”. The largest proportion of contribution to the total score was from “vocal symptoms” (48.98%). Consequently, vocal symptoms predominated in the sample.

The ThyPro-39 results are shown in Table 2.

**Table 2:** Results of the Thyroid-Related Patient-Reported Outcome-39 (ThyPro-39br) questionnaire, adapted to Brazilian Portuguese, in individuals who underwent total thyroidectomy 6 or more months before (n = 17)

	**Mean**	**Median**	**Standard deviation**	**Minimum- maximum**	**Interquartile range** **(Q_1_-Q_3_)**
**Goiter symptoms**	11.00	10.00	9.79	2-31	2.00-17.50
**Hyperthyroidism symptoms**	28.29	28.00	15.54	8-60	13.00-35.50
**Hypothyroidism symptoms**	24.94	25.00	18.64	0-50	9.13-43.88
**Eye symptoms**	31.29	25.00	20.65	8-89	14.00-41.50
**Fatigue**	70.12	67.00	27.40	25-100	50.00-100.00
**Cognitive problems**	51.47	52.00	31.85	1-95	21.00-80.50
**Anxiety**	54.47	56.00	26.59	10-96	30.00-75.00
**Depression**	48.53	45.00	25.45	7-97	29.00-58.50
**Emotional aspects**	55.53	60.00	25.52	1-95	32.00-68.00
**Impact on social life**	18.71	17.00	20.75	0-67	00-21.00
**Impact on daily life**	28.00	22.00	26.34	0-80	3.50-50.00
**Appearance**	27.59	21.00	25.81	1-80	1.00-47.00
**Overall quality of life**	29.41	25.00	34.50	0-100	00-50.00
**Final composite result**	49.28	51.00	19.65	19-77	31.19-69.00

Among the scales assessed by ThyPro-39, a predominance of high scores was observed in fatigue, followed by emotional aspects, anxiety, cognitive problems, and depression.

## DISCUSSION

This study aimed to analyze self-assessment of voice and swallowing and their impact on quality of life in the late postoperative period following total thyroidectomy. The results indicated that symptoms related to voice and swallowing are present in women who underwent total thyroidectomy more than 6 months before, and that the surgery negatively impacts quality of life, particularly regarding well-being and physical and mental function.

These data corroborate previous studies on symptoms persisting over time in up to 50% of cases, significantly impacting these patients’ quality of life^(3,6)^. The presence of symptoms verified with TVSQ was expected. In a study with 252 Brazilian women who had undergone thyroidectomy^(6)^, slightly more than half were more than 3 years post-operative, and their TVSQ scores were not different from those of women who had undergone surgery more recently. Thus, long-term symptoms, even in the absence of complaints, indicate that these women need to be monitored more closely and decisively during medical consultations, even 6 months post-surgery.

Management must be more attentive to persisting symptoms in the late postoperative period, given that individuals with thyroid disorders typically present with temporary symptoms that resolve between 3 and 6 months after thyroidectomy^(9,13)^.

The delicate innervation of the thyroid gland is closely related to laryngeal structures. Thus, vocal symptoms are common after thyroidectomy and are mostly transient^(2)^. Nonetheless, vocal symptoms contributed proportionally the most to the total score in this study. The literature indicates that patients may experience vocal fatigue, hoarseness, a deep voice, difficulty speaking loudly, a globus sensation, a dry throat, choking, and throat clearing following thyroid surgery^(5)^.

Although it contributed proportionally less to the final total TVSQ score, “oropharyngeal-laryngeal symptoms” achieved a relative score similar to that of “vocal symptoms”. The “oropharyngeal-laryngeal symptoms” domain encompasses aspects related to changes in swallowing physiology, which can cause throat clearing after swallowing, difficulty swallowing, choking, and a sensation of a lump in the throat^(5)^. These symptoms are often underestimated even in the immediate postoperative period and require closer attention in clinical practice, regardless of the postoperative time.

This study found that, in terms of the impact on quality of life, patients who underwent total thyroidectomy reported higher scores for fatigue symptoms and mental well-being, based on the sample’s responses. Fatigue is reported as an important measure of health-related quality of life^(8)^. A study with 281 patients with chronic fatigue following total thyroidectomy had reports of detrimental changes in daily life due to fatigue, establishing fatigue as a debilitating issue that impacts well-being^(14)^.

Hormonal changes, specifically postoperative fatigue, are a common adverse effect. Notably, a study involving 110 patients with thyroid cancer, 93% of whom underwent total thyroidectomy, found that more than half cited sudden episodes of exhaustion and reduced sleep quality as their main concerns regarding quality of life after surgery^(8)^.

Regarding the prevalence of these effects in the late period, a study found improved scores in the Brief Fatigue Inventory (BFI) at 6 and 12 months after surgery in patients who had undergone partial thyroidectomy, while it worsened in the same time points in the total thyroidectomy group^(15)^. This suggests that symptoms of fatigue and, consequently, of well-being also vary depending on the extent of the surgery performed. All patients in the present study underwent total thyroidectomy.

Thus, endocrinologists must supervise patients undergoing thyroidectomy to monitor and regulate their postoperative thyroid function and to advance the monitoring of cases from a physical and functional perspective. It is also important to consider long-term interprofessional support for patients’ mental health conditions.

The consequences of total thyroidectomy evidently extend beyond the immediate postoperative period, lasting longer than 6 months. Women who undergo this procedure frequently report the persistence of vocal and oropharyngeal-laryngeal symptoms. Such persistence has demonstrated its significant adverse impact, coupled with a decline in quality of life after surgery, affecting not only the patients’ physical well-being but also their emotional state.

These findings highlight the importance of carefully monitoring and continuously assessing vocal function and overall health status in patients undergoing total thyroidectomy. Furthermore, ongoing research in this area is essential for the development of more effective strategies for managing postoperative symptoms, aiming to improve the quality of life and satisfaction of patients facing the challenges that arise from this surgical intervention.

This study has some limitations. Among the main ones are possible selection biases and the absence of complementary instrumental tests for evaluating voice and swallowing. Moreover, it had a small sample, in which analyses beyond descriptive ones are not indicated. Studies with larger samples are necessary to establish and test hypotheses through inferential analyses. We should also note that the time interval between surgery and data collection in this study was highly variable and that changes in surgical techniques and care protocols often occur over time, which may have introduced a confounding factor.

## CONCLUSION

Women who undergo total thyroidectomy experience long-term vocal and swallowing symptoms. However, vocal symptoms associated with the negative impact of surgery on physical, emotional, and mental well-being are predominant. The most compromised aspects of quality of life relate to fatigue, emotional aspects, anxiety, cognitive problems, and depression.
