# Supplementary material for: Patient-reported voice and swallowing outcomes and their impact on quality of life in the late postoperative period following total thyroidectomy
Source: Codas. 2026 Jul 31;38(5):e20250387. doi: 10.1590/2317-1782/e20250387en (PMC13440682; doi:10.1590/2317-1782/e20250387en)
Supplement: Translation

# Translation: Autoavaliação da voz e deglutição e impacto na qualidade de vida no pós-operatório tardio de tireoidectomia total

**DOI:** 10.1590/2317-1782/e20250387pt

**Authors:** Lívia Lima do Nascimento Silva, Vivian Lisboa de Lucena, Ana Flávia de Sales Cândido, Raphaela de Lima Cruz, Anna Alice Almeida, Leandro Pernambuco

**Affiliations:** 1 Curso de Graduação em Fonoaudiologia, Universidade Federal da Paraíba – UFPB - João Pessoa (PB), Brasil.; 2 Departamento de Fonoaudiologia, Hospital Napoleão Laureano – HNL - João Pessoa (PB), Brasil.; 3 Programa de Pós-graduação em Modelos de Decisão e Saúde – PPGMDS, Universidade Federal da Paraíba – UFPB - João Pessoa (PB), Brasil.; 4 Curso de Graduação em Fonoaudiologia, Universidade Federal de Pernambuco – UFPE - Recife (PE), Brasil.

**Keywords:** Tireoidectomia, Transtornos de Deglutição, Disfonia, Pós-operatório, Qualidade de Vida

## Abstract

**Objetivo:**

Analisar a autoavaliação da voz e deglutição no pós-operatório tardio de tireoidectomia total e o impacto da cirurgia na qualidade de vida.

**Método:**

Trata-se de um estudo descritivo, observacional e transversal, aprovado pelo Comitê de Ética em Pesquisa da instituição. Participaram do estudo 17 mulheres com idades entre 32 e 58 anos, majoritariamente casadas, com ensino superior completo, residentes fora da região metropolitana submetidas à tireoidectomia total sem esvaziamento cervical, sem radioterapia adjuvante e sem acompanhamento fonoaudiológico pós-operatório há, no mínimo, 6 meses. Foram coletados dados demográficos, clínicos e os resultados dos questionários Thyroidectomy-Related Voice and Symptom Questionnaire (TVSQ) e Thyroid-Related Patient-Reported Outcome - 39 (ThyPro-39). A análise dos dados foi realizada de forma descritiva.

**Resultados:**

A mediana do escore total do TVSQ foi 26 e os escores mais elevados foram encontrados nos dois casos que fizeram esvaziamento cervical. O domínio 1 (sintomas vocais) do TVSQ foi o que contribuiu mais para o escore total (48,52%). No domínio 2 (sintomas orofaringolaríngeos) do TVSQ, os escores ficaram mais próximos do limite máximo, que representa maior frequência dos sintomas. Os resultados do ThyPro-39 revelaram maior impacto da tireoidectomia nos aspectos fadiga, ansiedade, problemas cognitivos, emoção e depressão.

**Conclusão:**

Mulheres submetidas à tireoidectomia total apresentam sintomas vocais e de deglutição, com predomínio dos sintomas vocais em longo prazo, associados ao impacto negativo da cirurgia no bem estar físico, emocional e mental. Além disso, há um comprometimento na qualidade de vida no que se refere à fadiga, aspectos emocionais, ansiedade, problemas cognitivos e depressão.

## INTRODUÇÃO

A tireoidectomia é a técnica cirúrgica mais comumente utilizada em casos de patologias que afetam a glândula tireoide, objetivando realizar a secção total ou parcial da estrutura, a depender do acometimento^(1)^. Em muitos casos, esses pacientes manifestam uma série de sintomas que afetam a voz e deglutição^(2)^.

A causa para o surgimento de alterações na voz e deglutição pode ser decorrente de danos aos nervos laríngeos, em especial, o nervo laríngeo recorrente (NLR) ou ramo externo do nervo laríngeo superior (NLS)^(3)^. Entretanto, também se observam outras possíveis etiologias para a condição conhecida como “síndrome pós-tireoidectomia”: trauma na cartilagem aritenoide; fixação laringotraqueal dos músculos envolvidos por aderências pós-operatórias, elevação reduzida da laringe, lesões do plexo nervoso peritireoidiano extrínseco; edema e trauma no músculo cricotireóideo^(2,4)^.

Em razão disso, estudos demonstram que a tireoidectomia pode levar ao comprometimento vocal e da funcionalidade do sistema orofaringolaríngeo^(5,6)^. Entre os principais sintomas, os mais relatados são: rouquidão, fadiga vocal, voz grave, dificuldade para falar em voz alta, sensação de corpo estranho na faringe, engasgo, pigarro durante a deglutição e garganta seca^(6)^.

Alguns estudos relatam que alterações vocais e de deglutição podem continuar persistindo, mesmo no momento tardio de tireoidectomia total^(3)^. Um estudo que possuía o objetivo de desenvolver evidências de validade baseadas na estrutura interna da versão em português brasileiro de um instrumento para identificação precoce e monitoramento desses sintomas, teve por maioria dos seus respondentes pós-operatório tardio e, ainda assim, tinham queixas consideráveis^(7)^.

Além das alterações de voz e deglutição, problemas na qualidade de vida dos pacientes vêm sendo frequentemente relatados^(8,9)^ como: medo da recorrência da doença, baixa qualidade do sono, perda do emprego, ataques repentinos de cansaço, exaustão física, medo de uma segunda operação, sensação globular (nó na garganta), dificuldades de deglutição e exaustão mental^(8)^.

Desse modo, é essencial que o acompanhamento e descrição dessas queixas sejam contempladas para que seja possível monitorar quais sinais e sintomas persistem no paciente pós-tardio, período considerado a partir dos 6 meses após o procedimento cirúrgico^(10)^.

Diante disso, o objetivo deste estudo é o de analisar a autoavaliação da voz e deglutição no pós-operatório tardio de tireoidectomia total assim como o impacto na qualidade de vida no pós-operatório tardio de tireoidectomia total.

## MÉTODO

Trata-se de um estudo descritivo, observacional e transversal, aprovado pelo Comitê de Ética em Pesquisa com Seres Humanos do Centro de Ciências da Saúde da Universidade Federal da Paraíba sob o número 6.018.537/2023. Todos os participantes foram informados sobre os objetivos, riscos e benefícios da pesquisa e assinaram o Termo de Consentimento Livre e Esclarecido (TCLE). A pesquisa foi realizada no Hospital Napoleão Laureano, um hospital sem fins lucrativos, mantido por uma fundação, classificado como Centro de Assistência de Alta Complexidade em Oncologia (CACON) e localizado em João Pessoa, Paraíba, Brasil.

Os critérios de inclusão foram: pessoas de ambos os sexos, com idade entre 18 e 59 anos, submetidas à tireoidectomia total com ou sem esvaziamento cervical há, no mínimo, 6 meses. Foram excluídos indivíduos submetidos previamente a qualquer outro tipo de cirurgia de cabeça e pescoço; pacientes com recidiva tumoral e/ou metástases à distância; pessoas em terapia adjuvante como quimioterapia/radioterapia/iodoterapia no momento da coleta; pacientes com dificuldades para responder comandos simples ou com alterações cognitivas ou neurológicas registradas em prontuário, percebidas pelos pesquisadores ou relatadas por acompanhantes.

Inicialmente foi realizada uma busca ativa nos prontuários de pacientes atendidos no serviço de Fonoaudiologia do hospital e selecionados aqueles que atendessem aos critérios de inclusão para convidá-los via contato telefônico. Além disso, usuários presentes no hospital para realização de consultas médicas e/ou fonoaudiológicas tiveram seus prontuários analisados e foram convidados a participar do estudo, caso fossem elegíveis.

Com base nos critérios de elegibilidade foram identificadas 32 pessoas elegíveis por meio da análise de prontuário. Dessas, 15 foram excluídas por ausência de resposta ao contato ou recusa em participar. Portanto, a amostra foi composta por 17 pessoas, todas do sexo biológico feminino, idades entre 32 e 58 anos (média: 46,64; DP: 8,65), que realizaram tireoidectomia total entre os anos de 2004 e 2022. A maioria das participantes era casada (n=12; 70,5%), com ensino superior completo (n=07; 41,1%), residentes de cidades de fora da região metropolitana (n=09; 52,9%), submetidas à tireoidectomia total sem esvaziamento cervical (n=14; 82,3%), sem radioiodoterapia ou radioterapia adjuvante (n=11; 64,7%), e sem fonoterapia prévia para voz e/ou deglutição (n=14; 82,3%).

A aplicação dos instrumentos de coleta foi feita por meio de duas modalidades: presencial ou digital, por meio da plataforma *Google Forms*. Em ambas as modalidades foi aplicado um *checklist* de elegibilidade e um formulário para coleta de dados demográficos e clínicos complementares aos dados colhidos previamente no prontuário. Em seguida, foram administradas as versões na língua portuguesa do Brasil dos questionários *Thyroidectomy-Related Voice and Symptom Questionnaire* (TVSQ)^(11)^ e *Thyroid-Related Patient-Reported Outcome - 39* (ThyPro-39br)^(12)^.

O TVSQ é um questionário organizado em 20 perguntas sobre sintomas de voz, deglutição e desconforto cérvico-torácico relacionados à tireoidectomia. Existem cinco possibilidades de resposta para cada item em uma escala do tipo Likert com valores crescentes de 0 a 4 (nunca, quase nunca, às vezes, quase sempre e sempre), correspondentes à percepção de frequência do sinal ou sintoma nos últimos trinta dias. Neste estudo, o TVSQ foi analisado de modo descritivo considerando os três domínios encontrados por Cândido (2021)^(7)^ ao analisar a validade estrutural do instrumento: 1) sintomas vocais; 2) sintomas orofaringolaríngeos e 3) sintomas de desconforto cervical e torácico. O segundo domínio do TVSQ, chamado “sintomas orofaringolaríngeos”, corresponde ao que se considera “sintomas de deglutição” neste estudo. O escore final do TVSQ é obtido pelo somatório simples de todos os itens e pode variar de 0 a 80, sendo pior quanto mais alto o escore. A versão em língua portuguesa do Brasil do TVSQ ainda não possui pontos de corte definidos, portanto, para fins de comparações interindividuais neste estudo descritivo, os autores usaram outros dois indicadores: 1) o escore relativizado do domínio [(escore do indivíduo no domínio / escore máximo do domínio) x 100]; e 2) proporção de contribuição do domínio no escore total [(escore do indivíduo no domínio / escore total do indivíduo) x 100].

O ThyPro-39 é um instrumento para avaliação e identificação da autopercepção do efeito de doenças tireoidianas na qualidade de vida. O questionário contém 39 perguntas distribuídas em 13 categorias, cujas respostas também seguem uma escala do tipo Likert de cinco pontos, considerando a percepção do paciente durante as últimas quatro semanas (sendo 0 igual a ''não'' e 4 igual a ''muito''). As categorias contemplam: quatro escalas de sintomas físicos; sete escalas sobre bem-estar e função física, mental e social; uma escala referente à aparência; e um item sobre o impacto na qualidade de vida em geral. Os resultados seguem um padrão de transformação específico que gera uma pontuação de 0 a 100, distribuída para cada uma das 13 escalas do instrumento. Assim, quanto maior a pontuação, maior o impacto na qualidade de vida.

A análise dos dados foi realizada de forma descritiva, por meio das frequências absolutas e relativas, considerando média, mediana, desvio-padrão e distância interquartílica.

## RESULTADOS

Os resultados referentes à aplicação do TVSQ estão na Tabela 1.

**Tabela 1:** Resultado da aplicação Th*yroidectomy-Related Voice and Symptom Questionnaire (TVSQ)* adaptado para a língua portuguesa do Brasil em pessoas submetidas à tireoidectomia total há seis meses ou mais (n=17)

	**Média**	**Mediana**	**Desvio-padrão**	**Mínimo-máximo**	**Distância interquartílica** **(Q25%-Q75%)**
**Escore total**	27,00	26,00	15,062	3-49	14,00-40,50
**Domínio 1 (D1)** *Sintomas vocais (10 itens)*	13,88	16,00	9,347	0-28	4,00-21,00
**Domínio 2 (D2)** *Sintomas orofaringolaringeos (5 itens)*	8,82	8,00	5,35	0-18	5,00-13,00
**Domínio 3 (D3)** *Desconforto cervical e torácico (5 itens)*	4,29	3,00	3,73	0-12	1,50-7,50
**Escore relativizado de D1** *(soma dos itens de D1/40)*	34,67	40,00	23,35	0-70	10,00-52,5
**Escore relativizado de D2** *(soma dos itens de D2/20)*	44,11	40,00	26,76	0-90	25,00-65,00
**Escore relativizado de D3** *(soma dos itens de D3/20)*	21,47	15,00	18,68	0-60	7,50-37,50
**Proporção de D1 no escore total** (soma dos itens de D1/escore total)*100	49,05	48,98	21,87	0-85,71	36,66-66,67
**Proporção de D2 no escore total** (soma dos itens de D2/escore total)*100	35,53	30,95	22,30	0-100	24,04-45,28
**Proporção de D3 no escore total (** soma dos itens de D3/escore total)*100	15,41	15,38	10,78	0-33,33	6,60-24,95

A mediana do escore total foi 26, sendo que os dois maiores escores (49 e 48) foram de duas participantes que realizaram a tireoidectomia total com esvaziamento cervical. No escore relativizado, a mediana indicou que nos domínios “sintomas vocais” e “sintomas orofaringolaríngeos”, o escore alcançou 40% do escore máximo que cada um desses domínios podia alcançar. Já a maior proporção de contribuição no escore total foi do domínio “sintomas vocais” (48,98%). Em virtude disso, houve um predomínio de sintomas vocais na amostra.

Os resultados referentes ao ThyPro-39 estão na Tabela 2.

**Tabela 2:** Resultado da aplicação *Thyroid-Related Patient-Reported Outcome - 39 (ThyPro-39br)* adaptado para a língua portuguesa do Brasil em pessoas submetidas à tireoidectomia total há seis meses ou mais (n=17)

	**Média**	**Mediana**	**Desvio - padrão**	**Mínimo-máximo**	**Distância interquartílica (Q25%-Q75%)**
**Sintomas de bócio**	11,00	10,00	9,79	2-31	2,00-17,50
**Sintomas de hipertireoidismo**	28,29	28,00	15,54	8-60	13,00-35,50
**Sintomas de hipotireoidismo**	24,94	25,00	18,64	0-50	9,13-43,88
**Sintomas oculares**	31,29	25,00	20,65	8-89	14,00-41,50
**Fadiga**	70,12	67,00	27,40	25-100	50,00-100,00
**Problemas cognitivos**	51,47	52,00	31,85	1-95	21,00-80,50
**Ansiedade**	54,47	56,00	26,59	10-96	30,00-75,00
**Depressão**	48,53	45,00	25,45	7-97	29,00-58,50
**Emocional**	55,53	60,00	25,52	1-95	32,00-68,00
**Impacto na vida social**	18,71	17,00	20,75	0-67	00-21,00
**Impacto no dia a dia**	28,00	22,00	26,34	0-80	3,50-50,00
**Aparência**	27,59	21,00	25,81	1-80	1,00-47,00
**Qualidade de vida em geral**	29,41	25,00	34,50	0-100	00-50,00
**Resultado composto final**	49,28	51,00	19,65	19-77	31,19-69,00

Dentre as escalas avaliadas pelo ThyPro-39, observou-se o predomínio de escores elevados em fadiga, seguidos pelos aspectos emocional, ansiedade, problemas cognitivos e depressão.

## DISCUSSÃO

Este estudo teve como objetivo analisar a autoavaliação da voz e deglutição e o impacto na qualidade de vida no pós-operatório tardio de tireoidectomia total. Os resultados indicaram que sintomas relacionados à voz e deglutição estão presentes em mulheres submetidas à tireoidectomia total há mais de seis meses e que a cirurgia provoca impacto negativo na qualidade de vida, em especial quanto ao bem-estar e à função física e mental.

Estes dados corroboram estudos anteriores acerca da persistência dos sintomas ao longo do tempo em até 50% dos casos, impactando significativamente a qualidade de vida desses pacientes^(3,6)^. A presença dos sintomas investigados por meio do TVSQ era esperada. Em pesquisa realizada com 252 mulheres brasileiras submetidas à tireoidectomia^(6)^, pouco mais da metade tinha mais de três anos de pós-operatório e o escore delas no TVSQ não foi diferente das mulheres submetidas à cirurgia há menos tempo. Portanto, a presença de sintomas no longo prazo, mesmo na ausência de queixas, indica ser imprescindível monitorar essas mulheres de modo mais atento e resolutivo nas consultas médicas mesmo após seis meses de pós-operatório.

A inclusão do gerenciamento mais atento à persistência dos sintomas no período tardio da cirurgia é importante visto que pessoas acometidas por doenças tireoidianas geralmente costumam apresentar sintomas temporários, que deixam de ser sintomáticos entre três e seis meses após a tireoidectomia^(9,13)^.

A delicada inervação da glândula tireóide mantém uma relação íntima com as estruturas da laringe. Assim, os sintomas vocais são frequentes após a tireoidectomia, e na maioria das vezes transitórios^(2)^. Entretanto, neste estudo os sintomas vocais foram os que mais contribuíram proporcionalmente no escore total. A literatura evidencia que após a intervenção cirúrgica na tireoide, os pacientes podem apresentar fadiga vocal, rouquidão, voz grave, dificuldade para falar alto, bolo faríngeo, garganta seca, engasgo e pigarro^(5)^.

Apesar de contribuir proporcionalmente menos no escore final total do TVSQ, o domínio “sintomas orofaringolaríngeos” alcançou um escore relativizado semelhante ao domínio “sintomas vocais”. O domínio “sintomas orofaringolaríngeos” compreende os aspectos pertinentes às alterações na fisiologia da deglutição, que podem provocar pigarro após deglutir, esforço para deglutir, engasgo e sensação de bolo na garganta^(5)^. Estes sintomas costumam ser subvalorizados até mesmo no pós-operatório recente e demandam um olhar mais criterioso na prática clínica, independentemente do tempo de pós-operatório.

Este estudo descobriu que, no que se refere ao impacto na qualidade de vida, pacientes submetidos à tireoidectomia total demonstram escores mais elevados de sintomas relacionados à fadiga e as condições de bem-estar mental a partir das respostas da amostra. A fadiga é relatada como uma importante medida de qualidade de vida relacionada à saúde^(8)^. Em um estudo com 281 pacientes com fadiga crônica após tireoidectomia total houve relato de alterações prejudiciais no cotidiano devido ao cansaço, configurando a fadiga como uma questão debilitante e que traz impacto ao bem estar^(14)^.

As alterações hormonais, especificamente o cansaço pós-operatório, consistem em um efeito adverso comum. Notavelmente, em uma pesquisa envolvendo 110 pacientes acometidos de câncer de tireoide, dos quais 93% foram submetidos à tireoidectomia total, constatou-se que mais da metade elencou episódios súbitos de exaustão e redução na qualidade do sono como suas principais preocupações relacionadas à qualidade de vida após a cirurgia^(8)^.

No que concerne à prevalência desses efeitos no período tardio, um estudo identificou que nos pacientes submetidos à tireoidectomia parcial houve melhorias nas pontuações do *Brief Fatigue Inventory* (BFI) nos períodos de 6 e 12 meses após a intervenção cirúrgica, enquanto no grupo submetido à tireoidectomia total houve piora nas pontuações do BFI nos mesmos intervalos de tempo^(15)^, sugerindo que os sintomas de fadiga e, consequentemente, de bem estar, também variam em função da extensão da cirurgia realizada. Neste estudo, todas as pacientes foram submetidas à tireoidectomia total.

Diante desse contexto, é fundamental que os pacientes que passam por tireoidectomia estejam sob a supervisão de um médico endocrinologista para monitorar e regular sua função tireoidiana pós-operatória e passem a avançar no monitoramento dos casos do ponto de vista físico e funcional. Somado a isso, é relevante considerar o apoio interprofissional no longo prazo ao paciente no que se refere às suas condições de saúde mental.

É evidente que as repercussões da tireoidectomia total vão além do período imediatamente após a cirurgia, estendendo-se para além de seis meses de pós-operatório. Nesse contexto, as mulheres submetidas a essa intervenção frequentemente relatam a persistência de sintomas vocais e orofaringolaríngeos. A persistência desses sintomas tem demonstrado seu impacto adverso significativo aliado à piora da qualidade de vida após a cirurgia, afetando não apenas o bem-estar físico, mas também o estado emocional dos pacientes.

Essas constatações destacam a importância do monitoramento cuidadoso e da avaliação contínua da função vocal e do estado de saúde geral em pacientes submetidos à tireoidectomia total. Além disso, a pesquisa em curso nessa área é fundamental para o desenvolvimento de estratégias mais eficazes de gerenciamento de sintomas pós-operatórios, visando melhorar a qualidade de vida e a satisfação dos pacientes que enfrentam os desafios decorrentes dessa intervenção cirúrgica.

Este estudo possui algumas limitações. Dentre as principais temos os possíveis vieses de seleção e a ausência de exames instrumentais complementares para avaliação da voz e deglutição. Além disso, trata-se de uma amostra pequena, na qual análises além da descritiva não são indicadas. Estudos com amostra maior serão necessários para que seja possível estabelecer hipóteses a serem testadas por meio de análises inferenciais. Também devemos destacar que o tempo entre a cirurgia e a coleta de dados neste estudo foi muito heterogênea e que frequentemente há mudanças relacionadas às técnicas cirúrgicas e protocolos assistenciais usados ao longo do tempo, o que pode ter sido um viés de confusão.

## CONCLUSÃO

Mulheres submetidas à tireoidectomia total apresentam sintomas vocais e de deglutição em longo prazo. No entanto, observa-se o predomínio de sintomas vocais associados ao impacto negativo da cirurgia no bem estar físico, emocional e mental. Os aspectos da qualidade de vida mais comprometidos referem-se à fadiga, aspectos emocionais, ansiedade, problemas cognitivos e depressão.
